# Salmonellosis Outbreak Traced to Playground Sand, Australia, 2007–2009

**DOI:** 10.3201/eid1807.111443

**Published:** 2012-07

**Authors:** Michael Staff, Jennie Musto, Geoff Hogg, Monika Janssen, Karrie Rose

**Affiliations:** New South Wales Health, Sydney, New South Wales, Australia (M. Staff, J. Musto);; Public Health Laboratory, Melbourne, Victoria, Australia (G. Hogg);; and Taronga Conservation Society Australia, Sydney (M. Janssen, K. Rose)

**Keywords:** Salmonella enterica variant Paratyphi B, communicable diseases, zoonoses, disease reservoirs, sand, bacteria, Australia, playground, bandicoot, bacteria, salmonellae, salmonellosis

## Abstract

A community outbreak of gastroenteritis in Australia during 2007–2009 was caused by ingestion of playground sand contaminated with *Salmonella enterica* Paratyphi B, variant Java. The bacterium was also isolated from local wildlife. Findings support consideration of nonfood sources during salmonellosis outbreak investigations and indicate transmission through the animal–human interface.

Variants of *Salmonella enterica* serovar Paratyphi B that use d-tartrate as a carbon source (known as *S. enterica* var. Java) primarily cause gastroenteritis. In contrast to experience in other countries (*1**–**4*), in Australia, *S. enterica* var. Java outbreaks have not been linked to food sources; the only outbreaks reported before 2007 were associated with imported ornamental fish (*5*). Sand in recreational sandboxes has been identified as a risk factor for infection of children with *S. enterica* serovar Typhimurium (*6*), and given the popular nature of this activity, there is a potential for sandboxes to pose a substantial public health hazard unless managed appropriately. We describe a protracted localized community outbreak associated with playground sand during 2007–2009, and highlight the need to consider nonfood sources when investigating salmonellosis outbreaks.

## The Study

In mid-2007, a routine review of serotypes among cases of *Salmonella* spp. infection reported to the New South Wales Health Department identified a probable *S. enterica* var. Java outbreak in a single local government area (population 57,000), and an investigation was initiated. We defined a case-patient as a person reported to health authorities during 2007–2009 who had had diarrhea for at least 24 hours, either lived in or had visited the local government area during the 7 days before the onset of diarrhea, and had provided a fecal sample from which *S. enterica* var. Java was isolated. Seventy-five case-patients were identified: ages ranged from 1 month through 60 years (median age 2 years, 10 months.); 34 were female. Three children were admitted to a hospital for 1–2 days; none died.

All isolates were sensitive to ampicillin, streptomycin, tetracycline, chloramphenicol, sulfathiazole, and spectinomycin. Results of phage typing by using standard techniques (*7*) were available for 74 isolates; 54 were classified as phage type Dundee, 19 as a uniform “reactions do not conform,” which closely resembled Dundee, and 1 as untypeable. Seventy-two isolates underwent multilocus variable number tandem repeat analysis (MLVA) typing (*8*); 69 were classified as 1-(12-17)-0-0-493 and 3 as distinctly different types. This MLVA type was unique to case-patients from this outbreak; it was not seen in isolates from other parts of New South Wales.

After an extensive investigation involving case-patient interviews and food and environmental sampling during 2007 and early 2008, exposure to playground sand at public parks and childcare centers was identified as the likely immediate source of the outbreak. *S. enterica* var. Java was isolated from 50 of 207 sand samples taken from 39 locations; no other *Salmonella* serotypes were detected. All 19 playgrounds with sandboxes from within the local government area were sampled, regardless of whether they were linked to case-patients. All 35 playground isolates came from 6 playgrounds that had received sand from a central depot within the past 12 months. *Salmonella* spp. were isolated from surface and deep (as far as 50 cm below the surface) sand samples. Despite multiple samples being taken, no isolates were obtained from the depot. Antimicrobial drug sensitivity testing, phage typing, and MLVA typing of isolates produced results indistinguishable from those of the outbreak strain. One contaminated playground was closed to human access, left undisturbed, and sequentially sampled every 3 months. Nine months passed before all samples taken from this playground were clear of the bacterium.

**Figure 1:**
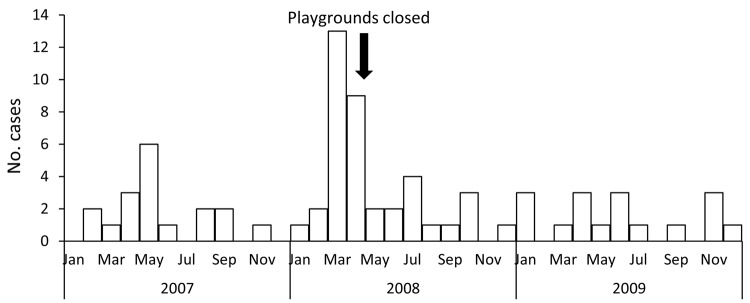
Number of cases of *Salmonella enterica* variant Java infection and month of onset in children playing in sandboxes, Australia, 2007–2009.

To confirm the hypothesis that sand from playgrounds contaminated with *S. enterica* var. Java was the immediate source of the outbreak, we performed an age-matched case–control study of case-patients 1 month to 4 years of age involving 16 case-patients and 32 controls during May 2008. Controls were selected from the registers of 2 local community child-health clinics. Exposure to playgrounds with contaminated sand within 7 days of symptom onset was associated with illness (matched odds ratio 3.7, 95% CI 1.1–12.1). In May 2008, the local authority began closing sandboxes, replacing the sand, and reopening. A substantial reduction in the number of case-patients reported occurred (Figure 1), although new case-patients continued to be reported throughout the study period.

**Figure 2:**
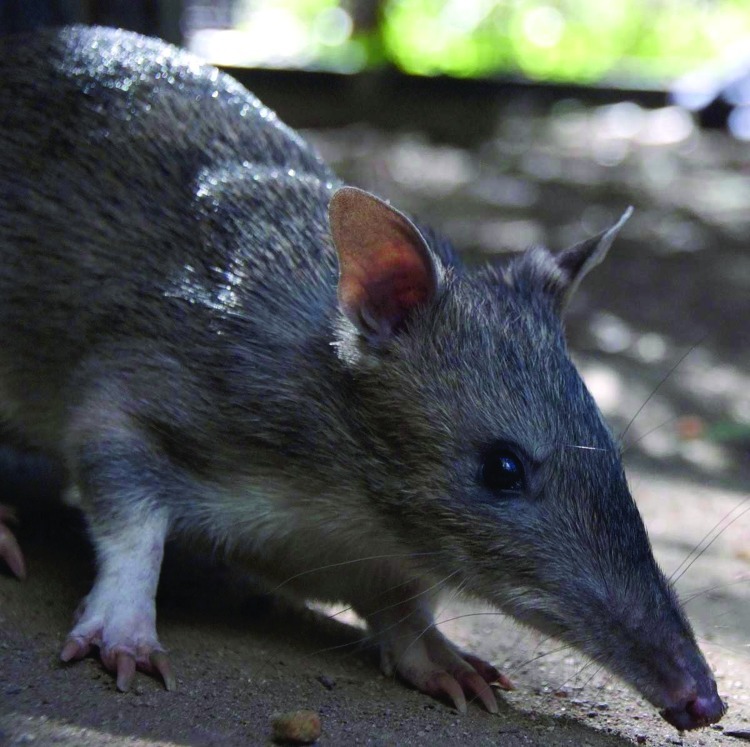
Long-nosed bandicoot (*Perameles nasuta*). Photograph courtesy of Taronga Zoo, Sydney, New South Wales, Australia.

To better characterize the distribution of the bacterium in the local ecosystems, we collected fecal and cloacal samples from 261 free-ranging animals of various species (Table). Thirty-four isolates of *S. enterica* var. Java were identified: most were from a marsupial species native to the local area, the long-nosed bandicoot, *Perameles nasuta* (Figure 2). Phage and MLVA types were indistinguishable from human and environmental isolates. The Australian Registry of Wildlife Health had no recorded evidence of disease associated with the isolation of *S. enterica* var. Java from long-nosed bandicoots (K. Rose, Taronga Conservation Society Australia, pers. comm.).

**Table:** *Salmonella enterica* var. Java isolates from animals in or near local government area, Australia, 1998–2009*

Species	Year	Reason for collection	Specimen type	No. animals tested	No. positive	MLVA (no.)
Bandicoot†	1998	Opportunistic screening	Feces	1	1	ND
Dog‡	2001	Unclear	Rectal swab	1	1	ND
Black duck	2008	Near contaminated playground	Feces from ground	5	2	1-16-0-0-493
Black rat	2008	Trapped in contaminated playground	Rectal swab	1	1	1-16-0-0-493
Bandicoot	2008	Trapped in contaminated playground	Cloacal swab	1	1	1-16-0-0-493
Brushtail possum	2009	Trapped in state park	Cloacal swab	11	1	1-14-0-0-493
Bandicoot	2009	Trapped in state parks	Cloacal swab and feces	67	18	1-13-0-0-493 (6), 1-14-0-0-493 (3), 1-15-0-0-493 (3), 1-16-0-0-493 (3), 1-17-0-0-493 (3)
Bandicoot	2008–2009	Wildlife rescue	Cloacal swab	12	2	1-13-0-0-493 (1), 1-15-0-0-490 (1)
Bandicoot	2009	Trapped in yard of a case-patient	Cloacal swab	3	2	1-16-0-0-493 (2)
Bandicoot§	2009	Wildlife rescue	Cloacal swab	3	3	01-13-0-0-493 (3)

*With the exceptions of samples from 1 bandicoot that reacted but did not conform (PT RDNC/A001), and 1 dog that was not tested, all phage types were Dundee. MLVA, multilocus variable number tandem repeat analysis; ND, not done. †Adult, sampled 10 km south of local government area. ‡Sampled10 km south of local government area. §Juvenile siblings.

The isolation of *S. enterica* var. Java from playground sand with the same phage type, MLVA pattern, and antibiogram as that of human isolates supported the hypothesis that ingestion of sand from playgrounds was the human exposure pathway for this outbreak. The case–control study found an association between infection in humans and exposure to at least 1 playground where an *S. enterica* var. Java isolate was found in the sand; these findings confirmed sand as the immediate source of infection. Although sand from the central depot was a common factor in all contaminated playgrounds where case-patients contracted the illness, the infection source for this facility remains unknown. It was located in a wild bushland setting, and it is feasible that transmission of the bacterium from local wildlife occurred.

## Conclusions

Native animals and wildlife have been implicated in previous community-wide outbreaks of salmonellosis (*9**,**10*). The isolation of *S. enterica* var. Java of common phage and MLVA types from human, animal, and environmental samples implies that the organism can survive within multiple ecosystems. The organism probably has multiple transmission pathways, involving interactions among humans, animals, and the environment. The persistence of the bacterium in playground sand for up to 9 months demonstrates that the organism can survive for a relatively long period in this environment and might provide information about the natural history of the bacterium and how it can infect humans.

We detected *S. enterica* var. Java with the outbreak phage type and MLVA typing in cloacal swab specimens from 31% of native long-nosed bandicoots sampled (Table); illness in the animals was not apparent. It is unclear whether the bacterium has a predilection for this species or whether the bandicoot exhibits a particular behavior that predisposes it to being colonized. Further research is merited.

The study identifies accidental sand ingestion as a previously unrecognized pathway for humans acquiring illness caused by *S. enterica* var. Java and provides further evidence for the need to manage this medium in playgrounds to protect public health. The emergence of human disease caused by *S. enterica* var. Java from this environmental source and the local colonization of wildlife with the same bacterium highlights the consequences of the interface between human and wildlife health. Further research and a more extensive systematic sampling program that includes the local government area that was the focus of this study are required to better understand the ecology of *S. enterica* var. Java and the potential role of wildlife in sustaining human disease outbreaks. Ultimate control of the outbreak might require a strategy that involves altering human behavior, the environment, and wildlife habitat.

## References

[1] Desenclos J, Bouvet P, Benz-lemoine E, Grimont F, Desqueyroux H, Rebiere I, Large outbreak of *Salmonella enterica* serotype *paratyphi B* caused by a goats’ milk cheese, France, 1993; a case finding and epidemiological study. BMJ. 1996;312:91–4. 10.1136/bmj.312.7023.91 8555937 PMC2349764

[2] Brown DJ, Mather H, Browning C, Coia J. Investigation of human infections with *Salmonella enterica* serovar Java in Scotland and possible association with imported poultry. Euro Surveill. 2003;8:35–40. 12631973 10.2807/esm.08.02.00399-en

[3] Ward L, Duckworth G, O’Brien S. *Salmonella* java phage type Dundee—rise in cases update. Euro Surveill. 1999;3:pii=1435.

[4] Denny J, Threfall J, Takkinen J, Lofdahl S, Westrell J, Varela C, Multinational *Salmonella Paratyphi* B variant Java (Salmonella Java) outbreak, August–December 2007. Euro Surveill. 2007;12:E071220.2.. 18179762 10.2807/esw.12.51.03332-en

[5] Musto J, Kirk M, Lightfoot D, Combs B, Mwanri L. Multi-drug resistant *Salmonella* java infections acquired from tropical fish aquariums, Australia, 2003–04. Commun Dis Intell. 2006;30:222–7. 16841504 10.33321/cdi.2006.30.18

[6] Doorduyn Y, Van Den Brandhof W, Van Duynhoven Y, Wannet W, Van Pelt W. Risk factors for *Salmonella* Enteritidis and Typhimurium (DT104 and non-DT104) infections in the Netherlands: predominant roles for raw eggs in Enteritidis and sandboxes in Typhimurium infections. Epidemiol Infect. 2006;134:617–26. 10.1017/S0950268805005406 16638166 PMC2870426

[7] Wang Q, Chiew R, Howard P, Gilbert G. *Salmonella* typing in New South Wales: current methods and application of improved epidemiological tools. N S W Public Health Bull. 2008;19:24–8. 10.1071/NB07036 18361865

[8] Lindstedt BA, Torpdahl M, Nielsen EM, Vardund T, Aas L, Kapperud G. Harmonisation of the multiple-locus variable-number tandem repeat analysis method between Denmark and Norway for typing *Salmonella* Typhimurium isolates and closer examination of the VNTR loci. J Appl Microbiol. 2007;102:728–35. 10.1111/j.1365-2672.2006.03134.x 17309622

[9] Ashbolt R, Kirk MD. *Salmonella* Mississippi infections in Tasmania: the role of native Australian animals and untreated drinking water. Epidemiol Infect. 2006;134:1257–65. 10.1017/S0950268806006224 16672107 PMC2870509

[10] Handeland K, Refsum T, Johansen BS, Holstad G, Knusten G, Solberg I, Prevalence of *Salmonella* Typhimurium infection in Norwegian hedgehog populations associated with two human disease outbreaks. Epidemiol Infect. 2002;128:523–7. 10.1017/S0950268802007021 12113498 PMC2869850

